# Long-term survival after tracheal replacement using a cryopreserved aortic allograft

**DOI:** 10.1016/j.xjtc.2024.08.015

**Published:** 2024-08-28

**Authors:** Geraud Galvaing, Emmanuel Martinod, Jean-Baptiste Chadeyras

**Affiliations:** a Department of Thoracic and Endocrine Surgery, Jean Perrin Comprehensive Cancer Center, Clermont-Ferrand, France; b Department of Thoracic and Vascular Surgery, Chirurgie thoracique et vasculaire, Assistance Publique des Hôpitaux de Paris, Hôpital Avicenne, Hôpitaux Universitaires Paris Seine-Saint-Denis, Université Sorbonne Paris Nord, Paris, France

**Figure:**
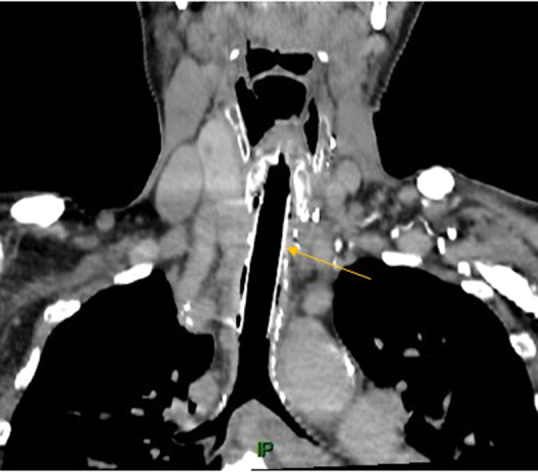
Postoperative image highlighting the silicone stent within the aortic allograft (*arrow*).

**Central Message**

Cryopreserved aortic allograft is a safe and valuable surgical option as a tracheal substitute. Stent removal is feasible and allows prolonged survival.

Resection of large tracheal segments remains a challenge for thoracic surgeons, particularly when end-to-end anastomosis cannot be performed despite the use of release maneuvers. The feasibility of tracheobronchial replacement using stented aortic matrices has been demonstrated.^1^^,^^2^ We report the case of a patient with prolonged survival after resection of an 8.5-cm tracheal segment, cryopreserved allograft replacement, and subsequent stent removal. Patient's informed consent for publication was obtained. Our institution's ethics committee approved the procedure (IRB 00013468; July 8, 2024).

## Case Report

A 67-year-old man was first admitted to our intensive care unit (ICU) in June 2014 for acute respiratory failure. His medical history included hypertension, atrial fibrillation, and stroke. He had previously undergone a tracheostomy for an unknown reason at another center. During that hospitalization, the tracheostomy was surgically recannulated to facilitate weaning from ventilator support. A diagnosis of compressive left goiter and surrounding tracheomalacia was also made. After 7 days in the ICU, the patient was discharged to a rehabilitation center with a tracheostomy tube but was subsequently lost to follow-up.

**Figure 1:**
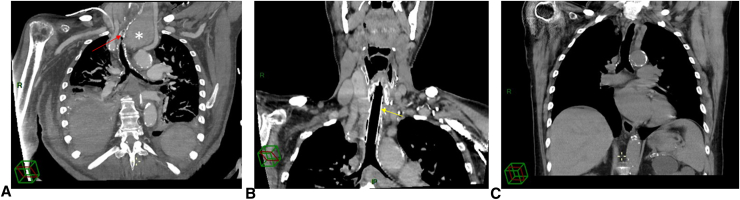
Coronal reconstruction of the trachea. A, Preoperative reconstruction showing the tip of the tracheostomy tube above the tracheal stenosis (*red arrow*) and the left thyroid lobe (*white star*). B, Postoperative reconstruction highlighting the silicone stent within the cryopreserved allograft (*yellow arrow*). C, Definitive postoperative view following stent removal, 1 year and 5 months after surgery.

**Figure 2:**
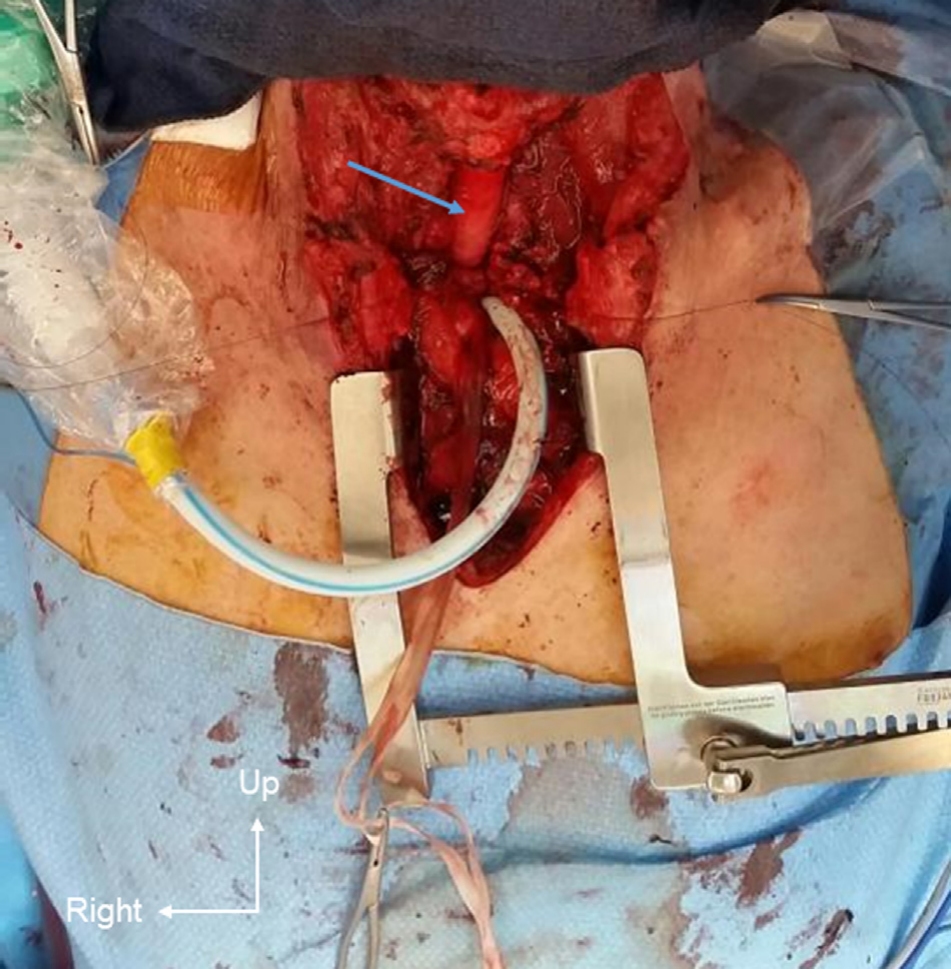
Operative view after completing the proximal and distal anastomoses, with the cryopreserved allograft indicated by the *blue arrow*.

During April 2015, the patient was readmitted to our ICU with acute respiratory failure and warfarin overdose. Computed tomography and bronchoscopy confirmed a 4-cm severe stenosis of the trachea, 3-cm distal to the vocal cords, with subsequent tracheomalacia and a known left compressive goiter (Figure 1, *A*). Due to the extent of the tracheal injury, end-to-end anastomosis was not possible, and the use of a cryopreserved allograft was proposed to the patient and his family. Through a collar incision associated with a partial sternotomy extended to the second intercostal space, we performed an extensive 8.5-cm tracheal resection and left hemithyroidectomy. A suprahyoid release maneuver allowed a 2-cm reapproximation of the upper trachea. A 5-cm cryopreserved aortic allograft was sewn in an end-to-end fashion using a 4-0 polydioxanone running suture on the membranous/posterior aspect and interrupted sutures on the cartilaginous/anterolateral aspects, and a 7-cm silicone stent (GSS stent; Novatech) was inserted (Figure 1, *B*). Both the graft and stent had the same diameter of 16 mm, determined based on the patient's tracheal diameter below the stenosis. Sternohyoid muscles were interposed as flaps over the proximal and distal anastomoses. Ventilation was performed via a field tracheotomy over the carina during the procedure, which was sutured before manubrium closure following reintubation through the stent (Figure 2). The patient was rapidly weaned from the ventilator, and his postoperative course was uneventful, without requiring bronchoscopy for sputum aspiration or granulomatous resection. Outpatient follow-up, including computed tomography scans, was initiated.

One year and 5 months later, the stent was easily removed because it interfered with the vocal cords (Figure 1, *C*). The patient lived a normal life, independent at home with his wife, without respiratory support until his death 5 years and 7 months after the allograft surgery, nearly 4 years after stent removal. The cause of death was unknown.

## Discussion

Cryopreserved aortic allograft is a reliable, available tracheal substitute for both malignant and benign conditions such as extensive tracheobronchial cancer, extensive thyroid cancer, and complex end-stage postintubation stenosis. Unlike other techniques,^3^ its use does not require multistep surgeries or immunosuppressive treatment, reducing the risk of postoperative infection. Stent removal is feasible several months or years after implantation, with the optimal time based on clinical and bronchoscopic assessment. De novo generation of pseudocartilage within the allograft has been demonstrated in vivo.^4^ Further studies are ongoing to better understand this phenomenon.^5^ Reporting this favorable experience with long-term survival is important as this innovative technique develops. It is important to note that the complexity of this procedure requires a high level of expertise and can only be performed at specialized centers.

## Conflict of Interest Statement

The authors reported no conflicts of interest.

The *Journal* policy requires editors and reviewers to disclose conflicts of interest and to decline handling or reviewing manuscripts for which they may have a conflict of interest. The editors and reviewers of this article have no conflicts of interest.
